# Trust in Medical Professionals Moderates Depression in Hong Kong during COVID-19

**DOI:** 10.1155/2023/9928793

**Published:** 2023-09-25

**Authors:** Daniel W. L. Lai, Jiahui Jin, Elsie Yan, Vincent W. P. Lee

**Affiliations:** ^1^Faculty of Social Sciences, Hong Kong Baptist University, Hong Kong; ^2^Wee Kim Wee School of Communication and Information, Nanyang Technological University, Singapore; ^3^Department of Applied Social Sciences, The Hong Kong Polytechnic University, Hong Kong

## Abstract

**Objectives:**

Given the continuing COVID-19 pandemic and the associated prevention and control measures implemented, the psychological burden brought by the pandemic on citizens is expected to increase. This study is aimed at exploring the predictors of depressive symptoms among Hong Kong people during the epidemic, as well as factors that could potentially alleviate the negative effects of the epidemic.

**Methods:**

The third wave follow-up survey (December 2021 to January 2022) from a longitudinal prospective survey study conducted in Hong Kong was used for a cross-sectional analysis. The participants (*n* = 803) are adults aged 18 and above in Hong Kong. Logistic and linear regression were performed to test the predictors and moderating effects, respectively, with depression as the outcome variable.

**Results:**

With minimized confounding effects of demographic variables, higher levels of concern about infection, experience with COVID-19 infection and previous epidemics, hassles, and trust in authority increased the odds of being depressed, while a higher level of trust in medical professionals reduced the odds of depression. Moreover, greater trust in medical professionals, as a moderator, lessened the positive associations between the levels of depression and hassles and concern about infection.

**Conclusions:**

Even though the threats of COVID-19 seem to have lowered, this study shows that a few factors associated with the pandemic continue to threaten people's mental health. However, developing greater trust in medical experts may be an effective way to relieve psychological burden.

## 1. Introduction

As of June 2022, more than 500 million positive cases of COVID-19 have been recorded by the World Health Organization. Despite having the longest life expectancy in the world, Hong Kong people have reported mental health as a concern [1]. With the outbreak of COVID-19, factors associated with the pandemic, such as worries about being infected, are found to be related to anxiety and depression among Hong Kong people [2]. Diverse health policy changes may have caused irreversible psychological trauma for people of all ages who reside in Hong Kong. In studies on the psychological influence of COVID-19 during its early stages, some scholars focused on the uncertainty and unpredictability of the virus, leading to panic and worries about being infected [3, 4]. Subsequently, the psychological impacts of the disease prevention measures, such as those of compulsory testing, quarantine, and social isolation, in addition to the hazards of the virus itself, began to be taken into account [5].

Fear of the virus was considered to be a stressor which imposed an adverse impact on people and resulted in a depressed mood [6]. For concerns about the coronavirus, infection fear was the vital predictor of the psychological burden brought by the pandemic [2]. Meanwhile, fear of infection was reported to be associated with perceived severity and risk [7]. Moreover, perceived severity and risk were partially caused by higher infection risk (e.g., surrounded by suspected/confirmed cases), and the experience with potential viral exposure increased psychological strain [8]. Other than the experience related to the present pandemic, the impact of previous epidemics, like SARS, has also been studied by researchers as a significant correlate [9].

Though fear of COVID-19 should diminish as the severity of the outbreak decreases, different psychological problems may have emerged, largely due to the strict disease prevention measures. In a cross-sectional comparison involving multiple countries, it was found that the higher the stringency of government response to the epidemic, the higher the levels of depression among citizens [10]. Even though more and more countries are choosing to coexist with the virus, the Hong Kong government was aiming to reach zero COVID and had introduced a series of preventive and control measures, such as launching the “LeaveHomeSafe” mobile phone application to track the visits of citizens. Considering Hong Kong's commitment to a zero-COVID policy, even isolated cases or minor outbreaks led to enhanced control measures. Toward the close of 2021, when a few positive COVID-19 cases had emerged, leading to a limited spread, the government had taken decisive action starting on January 7, 2022. The preventive measures were formulated by a team of expert consultants comprised of medical professionals from major local universities, but the final decision was made by the government. This included shutting down all entertainment venues like bars, halting dining-in at restaurants after 6:00 p.m., and making vaccination mandatory for accessing certain venues. Furthermore, medical experts openly urged the public to enhance adherence to preventive measures. However, this led to even more hassles in the lives of the public. There are significant population and regional differences in research on the effects of pandemic-related life hassles on depression. In previous studies, child caregivers (e.g., parents) who need to worry more about daily life have been the focus of research. Daily hassles were found to be a predictor of depressive symptoms [11]. One study in Germany showed that daily hassles were also negatively associated with mental health, regardless of subgroup characteristics [12]. Also, they proposed that the psychological impact brought by COVID-19 would be long-term. Thus, it is worth examining the effect of daily hassles at different stages of the pandemic, as well as in different regions.

Depression is one of the most frequently mentioned consequences of the psychological damage caused by epidemics, including SARS in 2003 [13]. Depressive symptoms have a negative impact on life quality and relationships [14]. Moreover, clinical research suggested that depression may cause drug dependence, which does great harm to patients' physical health [15]. When depressive symptoms are more severe, somatic symptom disorders may emerge, causing bodily symptoms [16]. In the past, many protective factors have been considered, among which public trust was widely believed to help people reduce psychological harm during the pandemic [17]. Based on this consensus, cultivating public trust was recommended to minimize the mental hazards brought by COVID-19 [18]. In Hong Kong, building public trust has been suggested as an approach to increasing vaccination uptake during the COVID-19 pandemic [19], while its protective role in reducing negative psychological outcomes has not been examined. Amid the COVID-19 pandemic, public trust emerged as a pivotal factor and was primarily focused on two key entities: governmental authorities and medical experts [19]. The governmental authority was responsible for public policy and crisis management, as medical experts provided scientific guidance on prevention, diagnosis, and treatment. While the preventive measures proposed by the Hong Kong government were largely based on and supported by medical expert advice, some independent medical professionals expressed concerns about the restrictiveness of these measures, potentially influencing public perception. Yet, little research has been available to identify the specific form of public trust that is more effective in mitigating adverse outcomes in pandemic situations. Therefore, this study is aimed at examining the role of public trust in pandemic response by differentiating the two forms of public trust (i.e., trust in authority and trust in medical professionals).

## 2. Methods

### 2.1. Research Design

A population-based, longitudinal prospective survey study was conducted with the general population in Hong Kong aged 18 and above. Quantitative telephone surveys were administered at three time points: T1 baseline (19 December 2020–6 January 2021, *n* = 1255), T2 follow-up (11 June 2021–21 July 2021, *n* = 1003), and T3 follow-up (21 December 2021–21 January 2022, *n* = 803). For this study, only cross-sectional data collected from the T3 follow-up wave were used, in which local outbreak of Omicron variant virus infection is on a rapid rise, and the government has announced tightening social distancing measures since January 7, such as closing public entertainment venues and restricting dine-in arrangements to daytime before 6 p.m. with a maximum of two people per table.

### 2.2. Data Collection

The inclusion criteria were Hong Kong residents at the age of 18 or above and able to communicate in Cantonese or Putonghua. A structured questionnaire was administered via telephone interview by a team of trained research assistants. The telephone numbers were randomly selected using a multistage procedure as applied in previous population-based telephone surveys in Hong Kong [20, 21]. To select the randomized telephone numbers for the calls, a local directory covering the prefixes of both the landline and mobile telephone numbers in Hong Kong was used. The prefixes randomly selected were used as “seeds,” for generating another set of numbers, using the “last digit plus/minus one/two” method, to form the second half of the telephone numbers for making the calls. After removing duplicate numbers, a total of 6000 telephone numbers randomly sequenced were used for further random selection. For telephone calls to a number with more than one eligible individual in the household, the last birthday method was adopted to identify the participant. Verbal informed consent was obtained from each of the participants. Research ethics approval was obtained from the Human Subject Ethics Subcommittee of the Hong Kong Polytechnic University (Approval Number: HSEARS20200814002), and verbal informed consent was obtained from each of the participants.

### 2.3. Measures

Depression is the dependent variable measured by the Chinese version of the 9-item Patient Health Questionnaire-9 (PHQ-9) [22]. The psychometric properties of the scale in Chinese populations were previously validated [22]. For each of the items, the participants were asked to rate their answers along a four-point scale ranging from 0 (not at all) to 3 (nearly every day). A higher total score, ranging between 0 and 27, indicates a higher level of depression. A total score of 0–4 indicates no depressive symptoms, 5–9 mild depressive symptoms, 10–14 moderate depressive symptoms, 15–19 moderately severe depressive symptoms, and 20–27 severe depressive symptoms [23]. A Cronbach's alpha coefficient of .961 was reported in this study. Many individuals tend to overlook mild depressive symptoms, thereby exacerbating the progression of the condition [24]. Therefore, it is imperative to accord due significance to early depressive symptoms to prevent the deterioration. Thus, a cut-off score of 5 and above was used to differentiate between the normal ones (0-4) and those with mild or higher levels of depression (5 and above) [23].

### 2.4. Independent Variables

The hassles brought by the COVID-19 pandemic referred to the level of disturbance experienced and was measured by asking the participants to indicate the level of disturbance experienced using a five-point scale ranging from 1 (none) to 5 (all the time). The 10 items covered daily areas related to leisure activities, economy, employment or study, social activities and interactions with family members, use of services, going out, travelling, and use of computer technologies, etc. A higher mean score along the scale range of 1 to 5 indicated a higher level of hassle experienced.

Awareness of the pandemic was represented by a few variables measuring the perceived severity toward the pandemic, level of concern toward the pandemic, perceived risk of being infected by COVID-19, current experience in contracting COVID-19, and previous experience of being infected in past pandemics.

The perceived severity of COVID-19 was measured by the items “COVID-19 is a severe disease.” Concern over COVID-19 was measured by the item “You are concerned about having COVID-19.” Current experience with COVID-19 was measured by three items: (1) “You have family member infected or suspected to be infected by COVID-19,” (2) “You have friends infected or suspected to be infected by COVID-19,” and (3) “Someone in the building you reside is infected or suspected to be infected by COVID-19.” Previous experience of being infected in past pandemics was measured by two items: (1) “You were infected by SARS, avian influenza, or swine flu” and (2) “You had family members or friends infected by SARS, avian influenza, or swine flu.” All items were rated along a 5-point Likert scale, ranging from “strongly disagree” to “strongly agree.” Perceived risk was measured by the question asking the participants to rate the chance that the participant himself/herself be infected by COVID-19 along a five-point scale ranging from “no chance at all” (1) to “very great chance” (5).

Two trust variables were included to represent trust in authority and trust in healthcare professionals in controlling the spread of COVID-19. The former was measured by two items asking the participants to rate their levels of trust in the politicians (e.g., legislators and political parties) and governmental officials (e.g., chief executive and secretaries) who were dealing with COVID-19, respectively. Trust in healthcare professionals was measured by one item for rating levels of trust in healthcare professionals (e.g., medical personnel and scientific experts) who were dealing with COVID-19. Each item was rated on an 11-point Likert scale, from 0 (lowest level of trust) to 10 (highest level of trust). Demographic variables examined included gender, age, the highest level of education, and economic activity status (i.e., being active or inactive). The questionnaire items used for measuring the dependent and major independent variables are illustrated in Appendix 1.

### 2.5. Data Analysis

SPSS 26.0 was used for data analysis [25]. The *p* values smaller than .05 were deemed statistically significant. Descriptive statistics are applied to show the characteristics and patterns of the demographic profile of participants and their responses. To examine the predictors of depression, a hierarchical logistic regression analysis was conducted. The outcome variable for all analyses conducted in the present study was depression, and the scores were grouped into being depressed (using the cut-off point of 5). The first blocks of independent variables included (i) demographic background (i.e., gender age, education attainment, and economic activity status); (ii) perceived severity of the COVID-19 pandemic, concern toward the COVID-19 pandemic, current experiences of COVID-19, previous experiences of other pandemics and epidemics, perceived risk of being infected, and hassles due to COVID-19; and (iii) trust in politicians and government officials and trust in medical professionals. Hierarchical linear regression was employed to assess the moderating effects of trust in authority and trust in medical professionals on the relationship between predictors and the PHQ-9 score. Subsequently, a simple slope test was performed using linear regression to further clarify these relationships. To avoid multicollinearity, the variables to be tested were centralized.

## 3. Results

### 3.1. Participants

Details of the participant's demographic characteristics are demonstrated in Table 1. Using the PHQ-9, 336 (41.8%) of the total participants were detected to be depressive based on the cut-off value of 5 and above, indicating a mild or higher level of depression. Table 1 also presents the association between the depression score and demographic variables. Being men, age 55 years and older, lower educational attainment, and inactive economic activity status were associated with a higher depressive score.

### 3.2. Factors Associated with Depression

As shown in the hierarchical logistic models in Table 2, educational attainment and economic activity status were found to be significantly related to being depressed in the first model. Participants with a lower educational level (OR = 1.876, 95% CI: 1.109~3.176) and active economic activity status (OR = .573, 95% CI: .417~.78) were less likely to be depressive.

The predictors related to awareness of COVID-19 (including severity, concern, previous experience, current experience, and perceived risk), hassles, and trust (in authority and medical professionals) were entered in the second model; economic activity status remained strongly significant (OR = .459, 95% CI: .308~.684, *p* < .001). Awareness related to pandemic concern (OR = 1.473, 95% CI: 1.184~1.832), awareness related to the current COVID-19 pandemic experience (OR = 1.411, 95% CI: 1.092~1.823), and awareness related to previous pandemic experience (OR = 2.493, 95% CI: 1.797~3.457) were the significant predictors of depression. Additionally, people who scored one point higher on hassles were 1.666 times (OR = 1.666, 95% CI: 1.284~2.161) more likely to be depressed. Moreover, participants who scored one point higher on authority trust are 1.130 times (OR = 1.130, 95% CI: 1.007~1.268) more likely to be depressive, while the odds of depression decreased by 11.4% (OR = .886, 95% CI: .792~.992) for a one-point increase in trust in medical professionals.

The pseudo-*R*^2^ values were .288 and .388 for the Cox and Snell *R*^2^ and Nagelkerke *R*^2^, respectively, illustrating an acceptable predictive ability by the final model. The significant contribution of predictors included in the second model to the overall model was evident with the improvement of the pseudo-*R*^2^ (see Table 2).

### 3.3. The Moderating Effect of Trust in Authority/Medical Professionals

Trust in medical professionals showed significant moderating effects on the association between PHQ-9 scores and hassles (△*R*^2^ = .003, △*F* = 4.925, *p* = .027), awareness related to concern (△*R*^2^ = .003, △*F* = 4.885, *p* = .027). Trust in authority did not show significant moderating effects in this study (see Table 3). Simple slope tests further showed that, for those who held less trust in medical professionals, the positive correlation between hassles and depressive symptoms was stronger than participants with greater trust in medical experts (see Table 4). Moreover, the positive association between the scores of depression and awareness related to concern was significant only for participants with a low level of trust in medical professionals (*B* = .803, *t* = 2.927, *p* = .004).

## 4. Discussion

The current research has identified the predictors of depression during the pandemic and the protective role of trust in medical professionals. Higher levels of concern about infection, current experience, previous experience, hassles, trust in authority, and lower levels of trust in medical professionals significantly predicted depressive symptoms, among which having previous pandemic experience (e.g., experiencing SARS and avian influenza) was the strongest predictor of depressive symptoms. Meanwhile, a high level of trust in medical professionals helped to mitigate the depressive symptoms reported by the pandemic.

Among all demographics, economic activity status was the only significant factor associated with depression. People who were economically active were about half as likely to suffer from depression as those who were not, which is in line with the findings in previous research. Ganson and Tsai suggested that more people have become unemployed due to the economic and employment downturns during COVID-19, leading to symptoms of anxiety and depression [26]. Though depressive symptoms differed significantly by gender, age group, and educational background both in previous studies and the results of the between-group test in the present research [27], the final model shows that the demographic effects were nonsignificant.

Concerns about the risks of being infected were still the vital predictor in the current study, which is consistent with the findings of previous studies [2]. Current experiences significantly predicted depression, a finding reported previously [9]. A noteworthy finding of this study was that people with experiences of epidemics, such as SARS and avian influenza, were more likely to be depressive than those without such experiences, which is different from a prior study [2]. Although past experience is considered part of psychological preparation [2], Yao et al. proposed that experiences of previous epidemics would adversely impact the current fear of infection based on the imprinting theory [9], and that such experiences not only served as a personal imprint but also affected family and friends around them [28]. Different from the results of previous research related to COVID-19, perceived severity and risk were not associated with depressive symptoms. As expected, in a long-term state of prevention and control, the impact on daily life (hassles) replaced the perceived threat of the virus itself as a significant predictor of depressive symptoms. Due to a higher vaccination rate than that in the previous months, the public has become less concerned about the virus due to the increasing perceived protection effect [29]. Meanwhile, an increase in depression may result from prolonged adherence to strict preventive measures [30].

Consistent with earlier findings, trust in medical professionals can reduce depressive symptoms [18]. Surprisingly, this study found a moderating effect of trust in medical professionals on the effects of hassles and concern about infection on depressive symptoms. However, though researchers reported that trust in government was negatively correlated with depression, anxiety, and psychological distress [17], the present study found that people with higher trust in authority were more likely to suffer from depression. The divergent impact of trust in medical experts on the population's depressive symptoms is attributed to rapidly changing government-enforced antipandemic prevention measures. Medical experts typically maintain consistent antipandemic recommendations, while governments must implement varying measures depending on the situation and take responsibility for virus transmission. As mentioned before, the questionnaires were collected at the beginning of the outbreak of the fifth wave (since late December 2021), and citizens' disappointment toward the resurging of the pandemic would probably affect the credibility of the political and government authorities. As one previous study reported, the degree of disappointment was a major reason for the variance of trust toward the government [31]. Herein, potential conflicts may have emerged due to the coexistence of high trust and disappointment at the same time, leading to self-inconsistency between belief and perceived emotion and the development of depressive symptoms [32].

## 5. Conclusions and Implications

This study indicates that having current and previous experiences of the pandemic, concern about being infected, and daily hassles are some important predictors of depression in the context of the COVID-19 pandemic. Among these predictors, a few could be the targets of change to curtail the negative mental health impact of the pandemic. The hassles experienced by citizens are probably the results of disease prevention and containment measures. Designing strategies to reduce hassles caused by measures and policies related to the prevention and containment of COVID-19 would be critical to further protecting the mental health of the population [33].

Concern about infection affects one's mental health, but preventive measures and policies, if working, should be able to better protect people from not just the virus but also the potential negative impact on their mental health. Yet, the concern may be better addressed by health professionals if messages on disease prevention and containment could reach the public in a more effective way. Despite the fact that hassles and concern about infection adversely affect mental health, trust in medical professionals serves to reduce the harmful mental health effects of the pandemic on people. Therefore, public health professionals should play a key role in further publicizing scientific findings, professional views, and recommendations related to the measures for handling the prevention of the spread of COVID-19. Using different mediums to disseminate relevant information was recommended to facilitate research translation [34]. Moreover, it is also suggested that the engagement of stakeholders, such as the government, is crucial to the successful translation of research findings into practice [34]. To further ease the negative psychological toll of the pandemic of the century, government officials could work more closely with healthcare professionals by providing means, channels, and support to translate professional public health knowledge into publicly understandable messages that could better help the citizens to overcome the impact of the pandemic on depression.

## Figures and Tables

**(a):** 

	Count (%)	PHQ-9 mean (SD)	*T*-test/*F*-test	Sig.
Gender				
Male	391 (48.7%)	6.68 (7.76)	2.162	.031
Female	412 (51.3%)	5.61 (6.18)		
Age				
18-34	175 (21.8%)	5.49 (6.88)	6.87	.001
35-54	266 (33.1%)	5.20 (6.19)		
55 and above	362 (45.1%)	7.13 (7.51)		
Educational attainment				
Lower secondary or below	262 (32.6%)	8.02 (7.97)	14.52	<.001
Upper secondary	355 (44.2%)	5.20 (6.07)		
Diploma/degree or above	186 (23.2%)	5.26 (6.74)		
Economic activity status				
Active	496 (61.8%)	5.57 (7.03)	-2.89	.004
Inactive	307 (38.2%)	7.04 (6.89)		

**(b):** 

	Mean (SD)
Awareness—perceived severity of COVID-19	4.27 (.80)
Awareness—concern over COVID-19	3.75 (.97)
Awareness—current experience with COVID-19	1.92 (1.13)
Awareness—previous experience with past pandemics	1.56 (1.08)
Awareness—perceived risk of being infected by COVID-19	2.96 (.97)
Hassles	3.37 (.68)
Trust in authority	4.79 (2.22)
Trust in medical professionals	6.06 (2.19)

**(a):** 

	Model 1		Model 2	
	B (SE)	Exp(*B*) 95% CI	B (SE)	Exp(*B*) 95% CI
Male^1^	.161 (.148)	1.174 (.879~1.568)	-.219 (.178)	.804 (.567~1.140)
Age	-.007 (.006)	.993 (.981~1.006)	-.012 (.008)	.988 (.973~1.003)
Education attainment				
Lower secondary or below^2^	.629⁣^∗^ (.268)	1.876 (1.109~3.176)	.331 (.316)	1.392 (.749~2.586)
Upper secondary^2^	.252 (.206)	1.286 (.858~1.928)	.307 (.243)	1.360 (.844~2.191)
Active economic activity status^3^	-.556⁣^∗∗∗^ (.163)	.573 (.417~.789)	-.778⁣^∗∗∗^ (.203)	.459 (.308~.684)

**(b):** 

	Model 2	
	*B* (SE)	Exp(*B*) 95% CI
Awareness—perceived severity of COVID-19	.268 (.138)	1.307 (.998~1.712)
Awareness—concern over COVID-19	.387⁣^∗∗^ (.112)	1.473 (1.184~1.832)
Awareness—current experience with COVID-19	.345⁣^∗∗^ (.131)	1.411 (1.092~1.823)
Awareness—previous experience with past pandemics	.913⁣^∗∗∗^ (.167)	2.493 (1.797~3.457)
Awareness—perceived risk of being infected by COVID-19	-.190 (.118)	.827 (.657~1.042)
Hassles	.510⁣^∗∗∗^ (.133)	1.666 (1.284~2.161)
Trust in authority	.122⁣^∗^ (.059)	1.130 (1.007~1.268)
Trust in medical professionals	-.121⁣^∗^ (.057)	.886 (.792~.992)

**(c):** 

	Model 1	Model 2
Cox & Snell *R*^2^	.031	.288
Nagelkerke *R*^2^	.041	.388

Note. ^1^Ref: female, ^2^ref: diploma/degree or above, ^3^ref: inactive economic activity status. ⁣^∗^*p* < .05, ⁣^∗∗^*p* < .01, and ⁣^∗∗∗^*p* < .001.

**Table 3:** Results of moderating effects with linear regression (dependent variable: PHQ-9 score).

Moderators	Predictors	△*R*^2^	△*F*	*p*
Trust in authority	Awareness—perceived severity of COVID-19	.001	1.321	.251
	Awareness—concern over COVID-19	.000	.004	.952
	Awareness—current experience with COVID-19	.001	1.115	.291
	Awareness—previous experience with past pandemics	.002	2.656	.104
	Awareness—perceived risk of being infected by COVID-19	.000	.264	.608
	Hassles (10 items)	.000	.041	.839

Trust in medical professionals	Awareness—perceived severity of COVID-19	.000	.345	.557
	Awareness—concern over COVID-19	.003	4.885	.027
	Awareness—current experience with COVID-19	.000	.083	.774
	Awareness—previous experience with past pandemics	.000	.680	.410
	Awareness—perceived risk of being infected by COVID-19	.000	.225	.635
	Hassles (10 items)	.003	4.925	.027

**Table 4:** Results of simple slope tests with linear regression (dependent variable: PHQ-9 score).

Moderators	Independent variables	Level of moderators	*B*	*t*	Sig
Trust in medical professionals	Hassles	Low	2.409	5.422	<.001
		Medium	1.792	6.394	<.001
		High	1.176	3.482	.001
	Awareness—concern over COVID-19	Low	.803	2.927	.004
		Medium	.413	1.935	.053
		High	.023	.082	.935

## Data Availability

Data will be available upon request.

## Abbreviations

PHQ-9: 9-item Patient Health Questionnaire-9.

## Supplementary Materials

Supplementary Materials Appendix 1: Measurements of dependent and major independent variables.
