# Supplementary material for: Trust in Medical Professionals Moderates Depression in Hong Kong during COVID-19
Source: Depress Anxiety. 2023 Sep 25;2023:9928793. doi: 10.1155/2023/9928793 (PMC11921831; doi:10.1155/2023/9928793)
Supplement: Supplementary Materials — Appendix 1: Measurements of dependent and major independent variables. [file 9928793.f1.docx]

**Appendix 1: Measurements of dependent and major independent variables**

**Depression: PHQ-9**

Over the last 2 weeks, how often have you been bothered by any of the following problems?

|  | Not at all | Several  days | More  than half  the days | Nearly  every  day |
| --- | --- | --- | --- | --- |
| 1. Little interest or pleasure in doing things | 0 | 1 | 2 | 3 |
| 1. Feeling down, depressed, or hopeless | 0 | 1 | 2 | 3 |
| 1. Trouble falling or staying asleep, or sleeping too much | 0 | 1 | 2 | 3 |
| 1. Feeling tired or having little energy | 0 | 1 | 2 | 3 |
| 1. Poor appetite or overeating | 0 | 1 | 2 | 3 |
| 1. Feeling bad about yourself — or that you are a failure or have let yourself or your family down | 0 | 1 | 2 | 3 |
| 1. Trouble concentrating on things, such as reading the newspaper or watching television | 0 | 1 | 2 | 3 |
| 1. Moving or speaking so slowly that other people could have noticed? Or the opposite — being so fidgety or restless that you have been moving around a lot more than usual | 0 | 1 | 2 | 3 |
| 1. Thoughts that you would be better off dead or of hurting yourself in some way | 0 | 1 | 2 | 3 |

**Hassles brought by the COVID-19**

Has the following aspect of your current life been affected by COVID-19?

|  | None | Sometimes | Regularly | Often | All the time |
| --- | --- | --- | --- | --- | --- |
| 1. Engaging in recreational activities | 1 | 2 | 3 | 4 | 5 |
| 1. Use of medical, social, and other public services | 1 | 2 | 3 | 4 | 5 |
| 1. Economic environment | 1 | 2 | 3 | 4 | 5 |
| 1. Employment/Work/Learning Environment | 1 | 2 | 3 | 4 | 5 |
| 1. Social Activities | 1 | 2 | 3 | 4 | 5 |
| 1. Interaction with family members | 1 | 2 | 3 | 4 | 5 |
| 1. Dining out | 1 | 2 | 3 | 4 | 5 |
| 1. Local tourism | 1 | 2 | 3 | 4 | 5 |
| 1. Overseas Travel | 1 | 2 | 3 | 4 | 5 |
| 1. Use of Information Technology (such as booking public services, shopping, communicating with others, etc.) | 1 | 2 | 3 | 4 | 5 |

**Awareness of pandemic**

|  |  | Strongly disagree | Slightly disagree | Neutral | Slightly agree | Strongly agree |
| --- | --- | --- | --- | --- | --- | --- |
| Perceived severity | 1. COVID-19 is a severe disease | 1 | 2 | 3 | 4 | 5 |
| Concern over COVID-19 | 1. You are very worried that you will contract COVID-19 | 1 | 2 | 3 | 4 | 5 |
| Current experience with COVID-19 | 1. Have any of your family members ever been confirmed or suspected to have contracted COVID-19 | 1 | 2 | 3 | 4 | 5 |
|  | 1. Have any of your friends ever been confirmed or suspected to have contracted COVID-19 | 1 | 2 | 3 | 4 | 5 |
|  | 1. Have there been any confirmed or suspected cases of COVID-19 in the building where you currently reside | 1 | 2 | 3 | 4 | 5 |
| Previous experience of being infected in past pandemics | 1. Have you ever been infected with SARS/ Avian Flu /Swine Flu | 1 | 2 | 3 | 4 | 5 |
|  | 1. Have your family members and friends ever been infected with SARS/ Avian Flu /Swine Flu | 1 | 2 | 3 | 4 | 5 |

|  |  | No chance at all | Small chance | Moderate chance | Considerable chance | Very great chance |
| --- | --- | --- | --- | --- | --- | --- |
| Perceived risk | 1. How likely do you think you are to get infected with COVID-19 | 1 | 2 | 3 | 4 | 5 |

**Trust in Authority**

1. How much do you trust Hong Kong’s political figures, such as legislators, political parties, and members of the Chinese People’s Political Consultative Conference, in handling COVID-19?

Please rate from 0 to 10, with 10 being complete trust and 0 being no trust at all.

1. How much do you trust Hong Kong government officials, such as the Chief Executive, Secretaries, etc., in handling the COVID-19 situation?

Please rate from 0 to 10, with 10 being complete trust and 0 being no trust at all.

**Trust in Medical Professionals**

How much do you trust medical professionals and scientific experts in dealing with COVID-19?

Please rate from 0 to 10, with 10 being complete trust and 0 being no trust at all.
